# Gene-gene interaction analysis identifies a new genetic risk factor for colorectal cancer

**DOI:** 10.1186/s12929-015-0180-9

**Published:** 2015-09-11

**Authors:** Jongkeun Park, Injung Kim, Keum Ji Jung, Soriul Kim, Sun Ha Jee, Sungjoo Kim Yoon

**Affiliations:** Department of Medical Lifesciences, The Catholic University of Korea, 505 Banpo-dong, Seocho-gu, Seoul, 137-701 Republic of Korea; Department of Epidemiology and Health Promotion, Institute for Health Promotion, Graduate School of Public Health, Yonsei University, Seoul, South Korea

**Keywords:** Gene-gene interaction, CRC, CDH13, *rs3865188*, *APN*SNPs

## Abstract

**Background:**

Adiponectin levels have been shown to be associated with colorectal cancer (CRC). Furthermore, a newly identified adiponectin receptor, T-cadherin, has been associated with plasma adiponectin levels. Therefore, we investigated the potential for a genetic association between T-cadherin and CRC risk.

**Result:**

We conducted a case–control study using the Korean Cancer Prevention study-II cohort, which is composed of 325 CRC patients and 977 normal individuals. Study results revealed that *rs3865188* in the 5’ flanking region of the T-cadherin gene (*CDH13*) was significantly associated with CRC (*p* = 0.0474). The odds ratio (OR) for the TT genotype as compared to the TA + AA genotype was 1.577 (*p* = 0.0144). In addition, the interaction between *CDH13* and the adiponectin gene (*APN*) for CRC risk was investigated using a logistic regression analysis. Among six *APN* single nucleotide polymorphisms (*rs182052*, *rs17366568*, *rs2241767*, *rs3821799*, *rs3774261*, and *rs6773957*)*,* an interaction with the *rs3865188* was found for four (*rs2241767*, *rs3821799*, *rs3774261*, and *rs6773957*). The group with combined genotypes of TT for *rs3865188* and GG for *rs377426* displayed the highest risk for CRC development as compared to those with the other genotype combinations. The OR for the TT/GG genotype as compared to the AA/AA genotype was 4.108 (*p* = 0.004). Furthermore, the plasma adiponectin level showed a correlation with the gene-gene interaction, and the group with the highest risk for CRC had the lowest adiponectin level (median, 4.8 μg/mL for the TT/GG genotype vs.7.835 μg/mL for the AA/AA genotype, *p* = 0.0017).

**Conclusions:**

The present study identified a new genetic factor for CRC risk and an interaction between *CDH13* and *APN* in CRC risk. These genetic factors may be useful for predicting CRC risk.

**Electronic supplementary material:**

The online version of this article (doi:10.1186/s12929-015-0180-9) contains supplementary material, which is available to authorized users.

## Background

Colorectal cancer (CRC) is the third most common cancer in men and the second among women worldwide [1]. While the CRC incidence rates are stabilizing or decreasing in historically-high risk countries, they are increasing in historically low-risk countries [2]. In Korea, the incidence rate of CRC has been continuously growing over the last 10 years, showing 7.0 and 5.3 % annual increases in men and women, respectively [3]. CRC is the third leading cause of cancer death in Korea, and is the only cancer with a mortality rate that has been increasing continuously for the past 10 years, while mortality rates for the top two cancers fallen gradually [3, 4].

The increase in the CRC incidence rate in Korea is thought to be associated with changes towards a Western diet and lifestyle which results in obesity. This is significant, as obesity has been reported to be associated with a high risk for CRC [5–7], and abdominal obesity was shown to be an independent risk factor for colon cancer as well [8]. In obese individuals, plasma adiponectin concentrations are low, and such levels have been shown to be associated with risk for CRC. This implies that decreased expression of adiponectin may be associated with increased risk for CRC development [9, 10].

Adiponectin, an abundant plasma protein exclusively expressed and secreted from adipocytes, modulates metabolic processes including the regulation of glucose levels and fatty acid oxidation. Through these mechanisms, adiponectin suppresses metabolic derangements that may result in type 2 diabetes (T2DM), obesity, atherosclerosis, non-alcoholic fatty liver disease, and metabolic syndrome. In fact, those with low plasma adiponectin levels are characterized by obesity and T2DM [11, 10]. The results from numerous studies have supported a relationship between plasma adiponectin levels and risk for CRC [12, 10, 13], and variants of the adiponectin gene (*APN*) have been shown to be associated with CRC risk [14, 15].

As an endogenous insulin sensitizer, adiponectin exerts its action through two G-protein coupled receptors, adiponectin receptor 1 (*AdipoR1*) and adiponectin receptor 2 (*AdipoR2*), which are expressed in colonic tissue [16, 17]. Adiponectin has been shown to suppress cell proliferation via activation of *AdipoR1* and *-R2* mediated 5' adenosine monophosphate-activated protein kinase (AMPK) in colon cancer cells [18]. Additionally, several polymorphisms of *APN* and *AdipoR1* have been shown to be associated with risk for CRC in various populations [19, 14, 20].

Recently, the third member of the adiponectin receptor family, *CDH13*, was found, and classified as a member of the cell surface glycoprotein family, functioning as a signaling transducer as well as a cell-cell adhesion molecule [21]. Loss of expression and aberrant methylation of the *CDH13* gene has been demonstrated in colorectal cancer [22, 23]. However, an association between the *CDH13* gene and CRC risk has yet to be described.

In the present study, we identified the nature of the association between *CDH13* and CRC, and found that the interaction between *APN* and *CDH13* was related to both the CRC risk and the plasma adiponectin level.

## Methods

### Ethics statement

Written informed consent was obtained from all study participants. The study was performed in accordance with the guidelines established by the Catholic Medical Center Office of Human Research Protection Program, and approved by the Institutional Review Boards of the Catholic University of Korea and Yonsei University.

### Study population

The study population was composed of 325 confirmed CRC patients from the Korean Cancer Prevention study-II (KCP-II), which included 200,595 individuals recruited from 16 health promotion centers nationwide. Patients were categorized as having CRC based on the International Classification of Diseases for Oncology (ICD-O) at the National Cancer Center of Korea [24]. As controls, randomly selected 1004 individuals with lack of CRC based on anamnesis and family history in the KCP-II were genotyped using Human SNP array 5.0 (Affymetrix, Santa Clara, CA, USA) and individuals with low genotyping call rates (<95 %) removed. And so were the ones with missing anthropometric measurement (SBP, DBP, waist circumference and BMI) or gender mismatch between genpotype and self-reported information. Thus, 977 normal individuals in the KCP-II were included for the present study [25] (Additional file 1: Figure S1). Genotypes of the control participants were previously determined using the same platform as was used in the present study [25]. Recorded and analyzed clinical characteristics of the participants included age, sex, systolic blood pressure (SBP), diastolic blood pressure (DBP), waist circumference (WC), body mass index (BMI), and plasma adiponectin level. Each participant was measured for weight and height while wearing light clothing, and BMI was calculated as mass (kg) divided by height in meters squared (m^2^). SBP and DBP were measured after a 15 min rest. Serum was separated from peripheral venous blood and the adiponectin level was measured using an ELISA kit following the manufacturer’s protocol (Mesdia, Korea) [26].

### Genotyping and single nucleotide polymorphism (SNP) selection

Genomic DNA isolated from the peripheral blood of participants was utilized for genotyping using the Human SNP Array 5.0 (Affymetrix, Santa Clara, CA, USA) at DNAlink Inc. (Seoul, Korea). For data accuracy, an internal quality control (QC) measurement was used: QC call rate (dynamic model algorithm) always exceeded > =86 %, and contrast QC <0.4. The heterozygosity of X chromosome markers identified the sex of each sample. Genotype calling was accomplished using the Birdseed (v2) algorithm. The genotype call rate for six *APN* SNPs (*rs182052*, *rs17366568*, *rs2241767*, *rs3821799*, *rs3774261,* and *rs6773957*) and one *CDH13 SNP* (*rs3865188*) were all above 95 %.

The single *CDH13 SNP* (*rs3865188*) was selected due to its previously identified association with plasma adiponectin levels [27]. In addition, all SNPs in *APN* on the chip were analyzed. The linkage disequilibrium (LD) between the *APN* SNPs was determined using Haploview 4.2 (Additional file 2: Figure S2).

### Statistical analysis

The *T*-test or *X*^*2*^ test was used to examine the differences in the distribution of demographic and clinical characteristics, as well as genotype frequencies between cases and controls. Hardy-Weinberg equilibrium (HWE) was also analyzed using the *X*^*2*^ test. A logistic regression analysis was used to evaluate the CRC risk associated with the genotype of each SNP, as well as the interactions between genotypes in comparison to control participants. The odds ratio (OR) and 95 % confidence intervals (CI) of CRC associated with *CDH13* and *APN*SNP genotypes was computed and adjusted for age, sex, and BMI. The *T*-test and analysis of variance test were used for normally distributed variables. Nonparametric tests included the Wilcoxon signed rank test and the Kruskal-Wallis test. All tests were considered statistically significant at *p* < 0.05. Statistical analyses were conducted using SAS software (ver. 9.2).

## Results

### General characteristics of study participants

The distribution of the demographic and clinical characteristics of the 325 CRC patients and 977 controls are shown in Table 1. The CRC patients were older with a greater proportion of males than the control group. They also had higher SBP, DBP, and BMI, and thicker WCthan controls. However, no difference in adiponectin levels was found between cases and controls.

**Table 1:** General characteristics of the study participants in Korean Cancer Prevention Study II (KCP-II)

	Total		*p*-value
	Normal participants (*n* = 977)	Colorectal caner patients (*n* = 325)	
Male(%)	56.5	72.9	
Age (year)	41.26 ± 8.44	52.82 ± 10.33	<.0001
SBP(mmHg)	120.66 ± 13.79	123.07 ± 16.43	0.011
DBP(mmHg)	73.76 ± 10.39	76.23 ± 11.51	<.0001
WC(cm)	81.30 ± 9.52	84.42 ± 8.79	<.0001
BMI(Kg/m^2^)	23.66 ± 3.12	24.17 ± 2.91	0.0095
Adiponectin(μg/ml)	8.33 ± 5.78	7.68 ± 4.85	0.3300

SBP; systolic blood pressure, DBP; diastolic blood pressure, WC; waist circumference, BMI; Body mass index

### Associations between SNPs and CRC

The genotype distributions of all SNPs were in HWE in the control group (Additional file 3: Table S1, *p* > 0.2). A significant association was found only between the *CDH13* SNP *rs3865188* and CRC; none of the six *APN* SNPs showed evidence of any association with CRC. The frequencies of *rs3865188* AA, AT, and TT genotypes were 0.4646, 0.3815, and 0.1538, respectively, in patients as compared to 0.4862, 0.4104, and 0.1034, respectively, in controls (*p* = 0.0474; Table 2). The logistic regression analysis revealed that the individuals with an *rs3865188* TT genotype had an increased risk of developing CRC as compared to the individuals with the other genotypes (AT or AA) in the recessive mode only (OR = 1.577, 95 % CI 1.095–2.272, *p* = 0.0144; Table 3). The risk estimate remained significant after adjustment for age and BMI, and showed a tendency toward risk even after an additional adjustment for sex (Table 3).

**Table 2:** Genotype distribution of *CDH13* and *APN* SNPs with respect to CRC in KCP-II

Gene	SNP	Group (n)	Genotype			*p*-value
			Frequency			
*CDH13*	*rs3865188*		AA	AT	TT	
		Case (325)	0.4646	0.3815	0.1538	0.0474 *
		Control (977)	0.4862	0.4104	0.1034	
*APN*	*rs182052*		AA	AG	GG	
		Case (325)	0.2646	0.5077	0.2277	0.4161
		Control (974)	0.2402	0.4979	0.2618	
	*rs1736656*		AA	AG	GG	
		Case(325)	0.0000	0.5540	0.9446	0.4235
		Control (977)	0.0100	0.3990	0.9591	
	*rs2241767*		AA	AG	GG	
		Case (325)	0.4954	0.4246	0.0800	0.7045
		Control (977)	0.5067	0.4023	0.0811	
	*rs3821799*		CC	CT	TT	
		Case (325)	0.1692	0.4677	0.3631	0.3357
		Control (968)	0.1395	0.4638	0.3967	
	*rs3774261*		AA	AG	GG	
		Case (324)	0.3179	0.5062	0.1759	0.1516
		Control (972)	0.3755	0.4743	0.1502	
	*rs6773957*		AA	AG	GG	
		Case (325)	0.3169	0.5046	0.1785	0.1417
		Control (977)	0.3756	0.4719	0.1525	

*: *P* < 0.05

**Table 3:** *rs3865188* of *CDH13* association analyses with risk of CRC in this study

		Unadjusted	Adjusted for age and BMI	Adjusted for age, sex and BMI
T-dominance	Point estimate	1.090	1.150	1.135
	95 % Confidence intervals	0.848–1.402	0.860–1.539	0.847–1.521
	*p*-value	0.5003	0.3451	0.3972
T-recessive	Point estimate	1.577	1.575	1.526
	95 % Confidence intervals	1.095–2.272	1.029–2.410	0.991–2.352
	*p*-value	0.0144*	0.0366*	0.0552

T-Dominance;TT + TAvs. AA T-recessive; TT vs. TA + AA, *: *P* < 0.05

### Gene-gene interaction for CRC risk

**Fig. 1:**
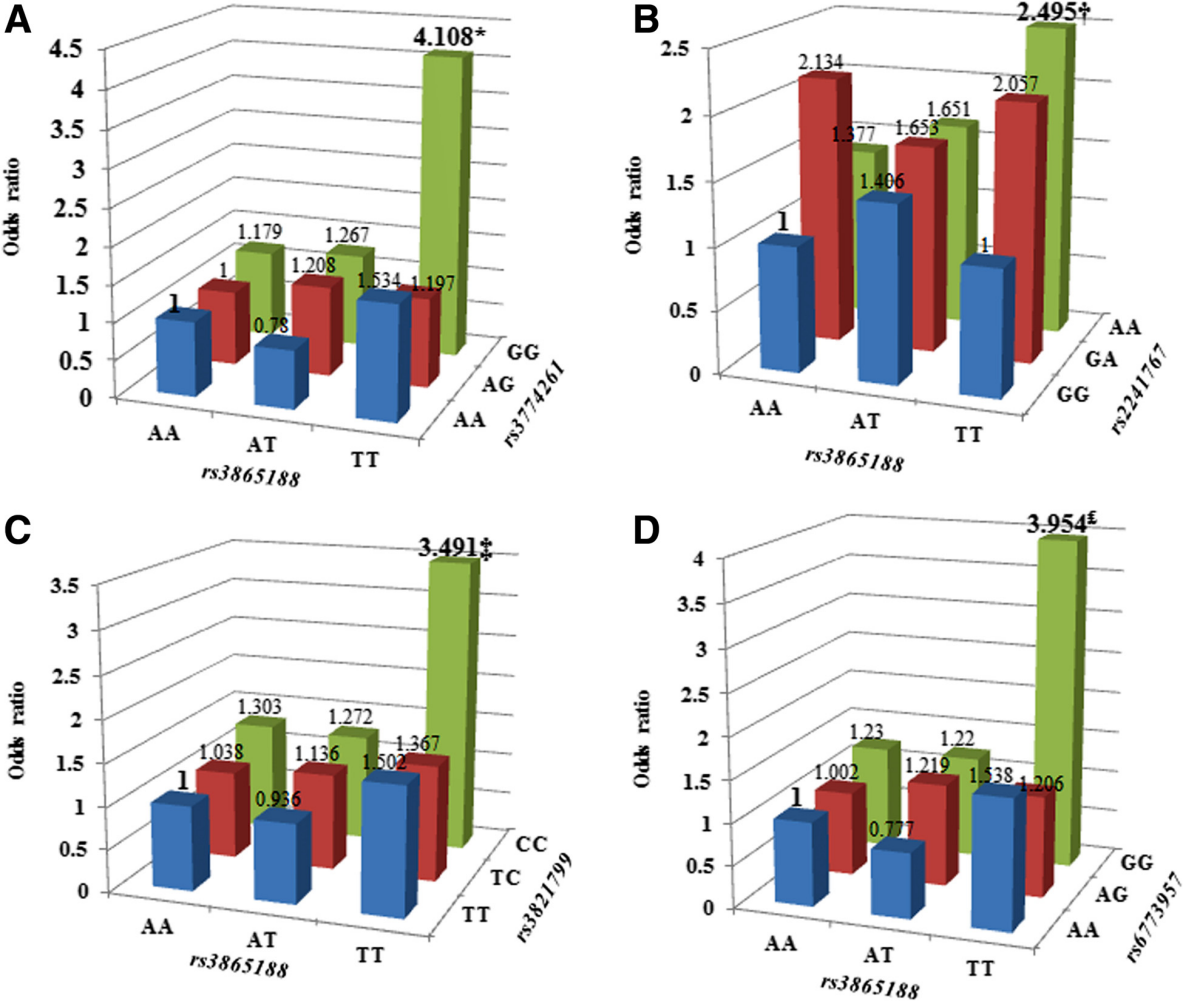
Adjusted odds ratios for colorectal cancer risk by interaction between *rs3865188* and each of the four *APN* SNP*s.* **a** *rs3865188-rs3774261* interaction, *; *p =* 0.004*,* **b**) *rs3865188-rs2241767* interaction, †; *p =* 0.0402, **c**) *rs3865188-rs3821799* interaction,‡; *p =* 0.0163, **d**) *rs3865188-rs6773957* interaction,₤; *p =* 0.0064. Adjusted for age, sex and BMI

Whether the presence of both variants could influence the risk for CRC was determined between *rs3865188* and each of the six *APN* SNPs. Among the six SNPs*,* fourshowed an interaction with *rs3865188* such that the combined genotypes were significantly associated with CRC risk. Those four SNPs, *rs2241767, rs3821799, rs3774261*, and *rs6773957,* formed an LD block (Additional file 1: Figure S1). Among individuals with an *rs3865188*TT genotype, a significant change in CRC risk was not observed for individuals who carried wild type alleles for all four SNPs when compared to controls. In contrast, among those with an *rs3865188* TT genotype, those whose genotypes were homozygous for the variant allele for each SNP had a much higher risk for CRC development as compared to the other genotype combinations (Fig. 1).

Individuals with the combined genotype of TT for *rs3865188* and GG for *rs3774261* had the most significantly increased CRC risk as compared to those with the other genotype combinations (Fig. 1a, OR = 4.108, 95 % CI 1.568–10.763, *p* = 0.004). Risks for CRC development due to interaction with *rs3865188* were also increased with *rs2241767* (Fig. 1b, OR = 2.495, 95 % CI 1.042–5.943, *p* = 0.0402), *rs3821799* (Fig. 1c, OR = 3.491, 95 % CI 1.259–9.679, *p* = 0.0163), and *rs6773957* (Fig. 1d, OR = 3.954, 95 % CI 1.471–10.629, *p* = 0.0064). However, *rs182052* and *rs17366568* were neither associated with CRC independently nor interacted with *rs3865188* for CRC risk.

### Correlation of the combined genotype with the adiponectin level

Because *rs3865188* was shown to be associated with the plasma level of adiponectin, and low levels of adiponectin were reported to be related to CRC risk [27], we investigated whether an association with CRC by gene-gene interaction was correlated with the plasma adiponectin level. The plasma adiponectin level was analyzed using nonparametric tests.

**Fig. 2:**
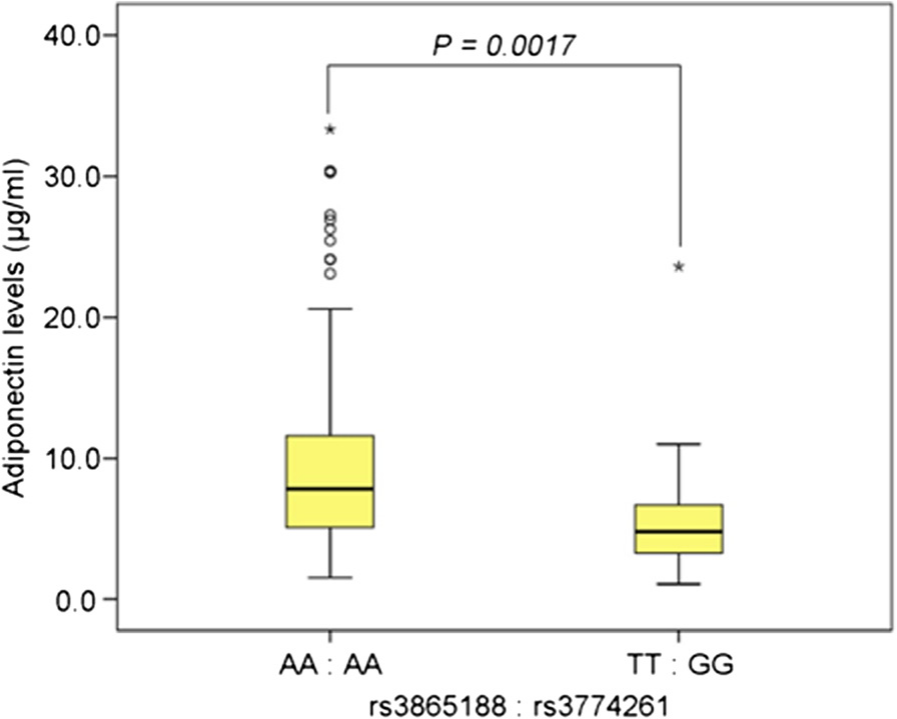
Difference in the plasma adiponectin level based on gene-gene interaction in KCP study-II. The median plasma adiponectin levels were7.835 and 4.800 μg/ml for individuals with the genotype combination of *rs3865188*:AA/*rs3774261*:AA and *rs3865188*:TT/ rs3774261:GG, respectively

The plasma adiponectin level was significantly lower in the groups with the risk genotype combination than in those with the other genotype combinations for all SNP pairs. Individuals with a TT genotype for *rs3865188* and a GG genotype for *rs3774261* had the lowest plasma adiponectin levels as compared to those with the AA genotype for *rs3865188* and the AA genotype for *rs3774261* (Fig. 2, *rs3865188*:AA/*rs3774261*:AA median = 7.83 μg/ml, *rs3865188*:TT/rs3774261:GG median = 4.80 μg/ml, *p* = 0.0017). A similar pattern was seen in the pairs of other genotype combinations (Additional file 3: Table S2).

## Discussion

This is the first study to examine gene-gene interactions between genetic variants of adiponectin and its new receptor, *CDH13*, with reference to CRC. Additionally, the present study also demonstrated the significant gene-gene interactions between*CDH13 rs3865188* and *APN* SNPs in a LD block in relation to the risk for CRC, with the combination of the *rs3865188* TT and *rs3774261* GG genotypes having the highest risk. Of interest, this risk increase was consistent with low adiponectin levels.

Adiponectin and its pathway have been implicated in the carcinogenesis of multiple cancers including CRC [28]. Adiponectin has been associated with metabolic syndrome, T2DM, and obesity [29–31], which are known risk factors for CRC [7, 32]. The results from several studies have indicated that adiponectin mRNA and plasma levels were decreased in patients with obesity and T2DM [33, 34], implicating adiponectin as a key player in the development of obesity and insulin resistance. Because this characteristic of adiponectinis attributable to carcinogenesis, the roles of adiponectin and its receptors have been studied in carcinogenesis [35–37].

*APN* SNPs have been controversial in CRC carcinogenesis association studies, as no single SNP has been consistently associated across the different studies [38–40]. While the *APN* gene was found not to be associated with CRC in the UK and US (New York) [38, 39], contrary results were found in a Chinese population [38]. The most recent large meta-analysis on the subject revealed that *rs2241766* alone showed an association with CRC in a specific genetic mode. In our study, none of the six *APN* SNPs were associated with either CRC risk or adiponectin levels. The *rs3774261* and *rs6773957APN* SNPs were shown to be associated with adiponectin levels in an American population [41]. Furthermore, *rs1063538*, which is in LD with these two SNPs was shown to have an association with CRC risk in a Chinese population [40]. The *rs3774261* and *rs6773957* SNPs had lower *p*-values than the other four SNPs, however, no associations with CRC risk or adiponectin levels were found in our study. Instead, we documented that these SNPs showed gene-gene interactions with *rs3865188* for CRC risk and adiponectin levels. Thus, a replication study with larger sample is required to clarify the nature of the relationship between *APN* variants and CRC risk in Koreans. Interestingly, four SNPs (*rs2241767, rs3821799, rs3774261*, and *rs6773957)* of the six *APN* SNPs formed an identical LD block in the Eastern Asians including Korean population, CHB; Han Chinese in Bejing, China, JPT; Japanese in Tokyo, Japan, whereas those did not in Caucasians such as CEU; Utah Residents with Northern and Western European Ancestry (HapMap project). Furthermore, it has been shown that rs3865188 is also highly associated with plasma APN levels in Filipinos, and Japanese as well [42, 43]. Thus, there is a possibility that gene-gene interaction between a *CDH13* SNP and adiponectin SNPs may affect East Asians and a further study is required to clarify this relationship.

CRC risk is mediated through *APN* receptors, as well as through *APN*. The results of a previous study using classification and regression tree analysis indicated that an environmental factor was associated with risk for CRC based on the interaction between *rs1063538* of *APN* and *rs1539355* of *AdipoR1* [40]. Results from an additional study demonstrated that adiponectin mediated AMPK activity via *AdipoR1* and *AdipoR2* in a colon cancer cell line [18]. AMPK plays critical roles in the regulation of glucose metabolism, insulin sensitivity, and lipid metabolism [44], all of which are correlated with tumorigenesis in humans [45]. Additionally, polymorphisms related to insulin resistance and obesity have been reported to be associated with CRC risk [46]. Thus, we anticipated that a receptor gene of *APN* may play an important role in the carcinogenesis of CRC.

Although we observed a marginal association of a *CDH13* polymorphism and no association of *APN* polymorphisms with CRC, the interaction of these SNPs showed significant associations with the risk of CRC, suggesting that *APN* function via *CDH13* should be considered in regard to CRC risk.

CDH13 expression was found in adult brain, lung, heart and blood vessels [47] and shown to be modulated by various factors such as FGF-2 [48], oxidative stress [49], progesterone, estradiol and serum factors [50], and zeb1 [51]. However, CDH13 expression in different ethnic groups has not been currently known.

The biological function of *CDH13* remains largely unknown. However, it is a known *APN* receptor, and binding of the high molecular weight form of *APN* to *CDH13* transduces signals and activates AMPK activity [44]. Furthermore, *CDH13* is known to function as a tumor suppressor gene, and suppression of *CDH13* expression by aberrant methylation in the CpG island of its promoter has been reported in CRC [23, 52] and other types of cancers [53–55]. Thus, we suggest that pathological regulation affected by the interaction between *CDH13* and *APN* may be mediated through AMPK in CRC.

Epidemiologic studies have identified sexual dimorphism in CRC risk, with a higher incidence and earlier onset in men [56] and female hormones have been suggested to play a protective role in CRC development [57]. Interestingly, we observed that *rs3865188*of *CDH13*was significantly associated with CRC risk in male (*p* = 0.049) but not in female (*p* = 0.880). Recently, circulating T-cadherin and high molecular weight adiponecin levels were shown to be negatively associated in angiographically stable coronary artery young male patients but not in female patients [58]. Therefore, male-specific CDH13 association in CRC risk could have a sex-specific contributing factor(s) involved. Clearly, further study with larger number of participantsis required to identify an underlying factor for this observation.

The present study was not without limitations. First, our sample size was limited. We selected 325 CRC patients and 977 normal individuals from the KCP-II. Further study will be needed in order to analyze more Koreans and members of other ethnic groups. Second, our study only identified interactions between genetic factors with respect to CRC risk, and therefore we did not investigate relationships between genetic factors and clinical information (cancer staging, drug treatment, other disorder). Additional studies will be required in order to more fully elucidate the nature of the correlations between clinical information, CRC, and gene-gene interactions.

## Conclusion

Nevertheless, we found a new gene-gene interaction with respect to CRC risk is uncovered, and identified a correlation between adiponectin level and the combined genotype. Our study suggests that gene-gene interaction between a *CDH13* SNP and adiponectin SNPs is a CRC risk genetic marker for predisposition in Korean population.

## Additional files

Additional file 1: Figure S1. Overall study population design. (TIFF 1061 kb) Additional file 2: Figure S2. Linkage disequilibrium of six SNPs of the adiponectin gene (*APN*). (TIFF 599 kb) Additional file 3: Table S1. Hardy-weinberg equilibrium (HWE) in the control group. **Table S2.** Differences on the adiponectin level in KCP-II based on the genotype combination between rs3865188- rs2241767, rs3865188- rs382179 and rs3865188- rs6773957. (DOCX 18 kb)

## Abbreviations

CRC: Colorectal cancer
SNP: Single nucleotide polymorphism
KCP-II: Korean Cancer Prevention study-II
*CDH13*: T-cadherin gene
*APN*: Adiponectin gene
OR: Odds ratio
95 % CI: 95 % confidence intervals
LD: Linkage disequilibrium
HWE: Hardy-Weinberg equilibrium
ICD-O: The International Classification of Diseases for Oncology
QC: Quality control
SBP: Systolic blood pressure
DBP: Diastolic blood pressure
WC: Waist circumference
BMI: Body mass index
T2DM: Type 2 diabetes
*AdipoR1*: Adiponectin receptor 1
*AdipoR2*: Adiponectin receptor 2
AMPK: 5' adenosine monophosphate-activated protein kinase
