# Supplementary figures and images for: Gene-gene interaction analysis identifies a new genetic risk factor for colorectal cancer
Source: J Biomed Sci. 2015 Sep 11;22(1):73. doi: 10.1186/s12929-015-0180-9 (PMC4566297; doi:10.1186/s12929-015-0180-9)

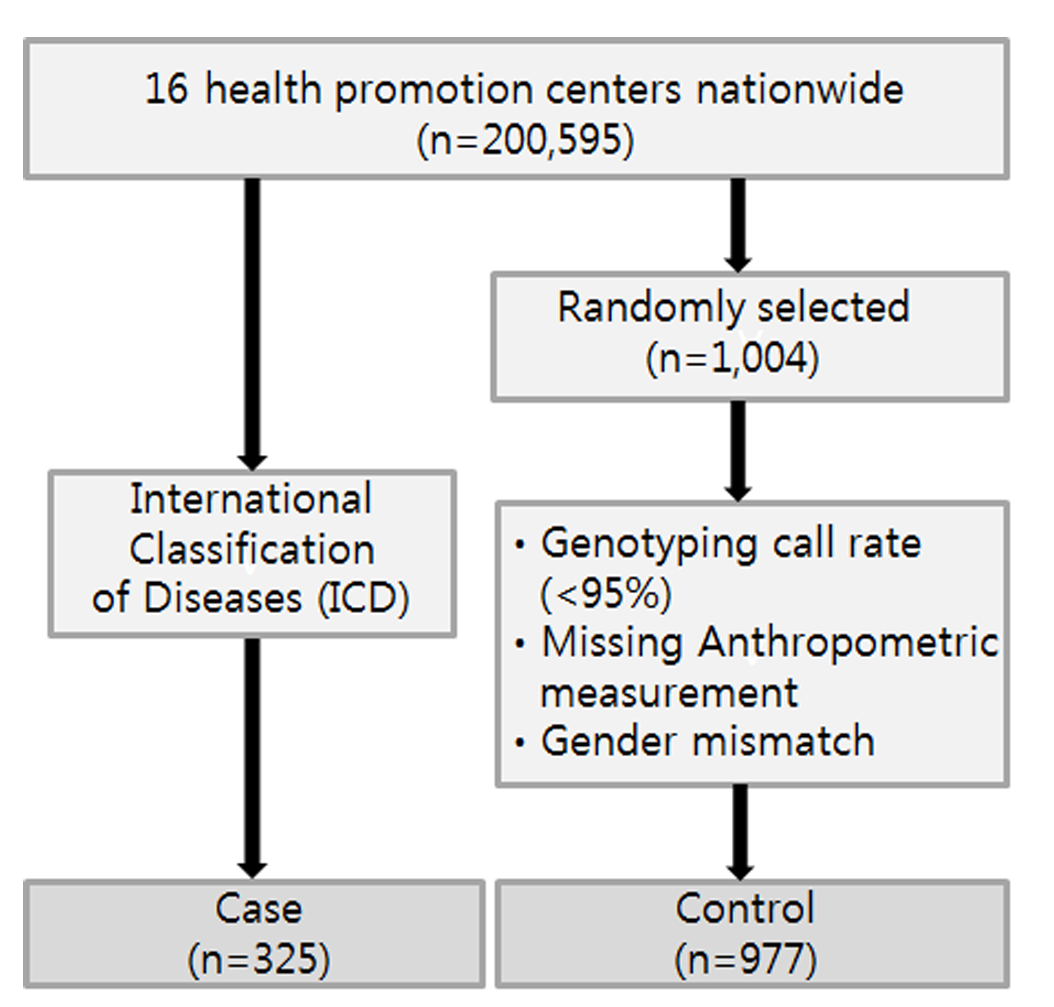

Supplement: Additional file 1: Figure S1. — Overall study population design. (TIFF 1061 kb) [file 12929_2015_180_MOESM1_ESM.tif]

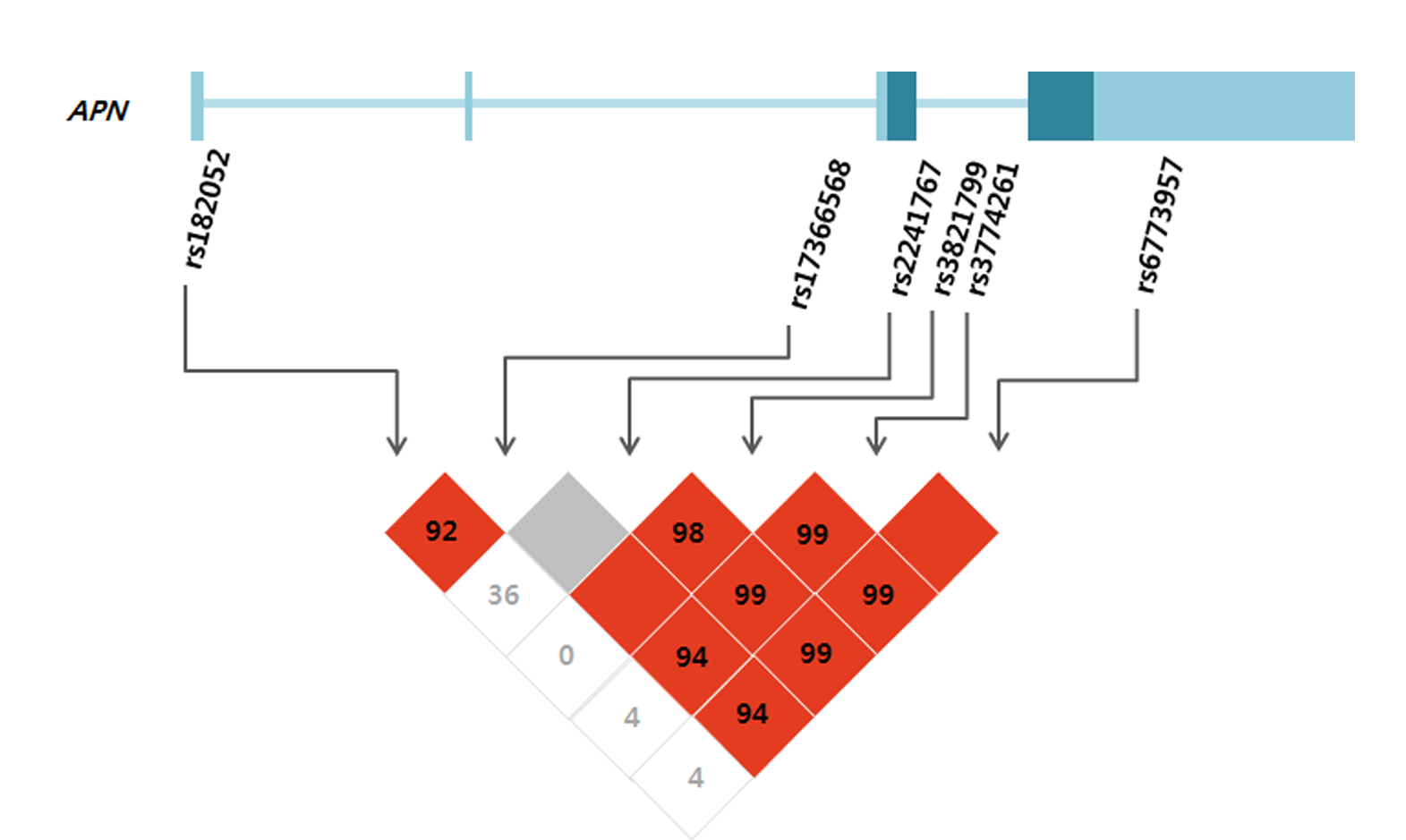

Supplement: Additional file 2: Figure S2. — Linkage disequilibrium of six SNPs of the adiponectin gene (APN). (TIFF 599 kb) [file 12929_2015_180_MOESM2_ESM.tiff]
