# Supplementary material for: Gene-gene interaction analysis identifies a new genetic risk factor for colorectal cancer
Source: J Biomed Sci. 2015 Sep 11;22(1):73. doi: 10.1186/s12929-015-0180-9 (PMC4566297; doi:10.1186/s12929-015-0180-9)
Supplement: Additional file 3: Table S1. — Hardy-weinberg equilibrium (HWE) in the control group. Table S2. Differences on the adiponectin level in KCP-II based on the genotype combination between rs3865188- rs2241767, rs3865188- rs382179 and rs3865188- rs6773957. (DOCX 18 kb) [file 12929_2015_180_MOESM3_ESM.docx]

Table S1. Hardy-weinberg equilibrium (HWE) in the control group.

|  | Frequency | | | | | | | |
| --- | --- | --- | --- | --- | --- | --- | --- | --- |
|  | | rs3865188 | rs182052 | rs17366568 | rs2241767 | rs3821799 | rs3774261 | rs6773957 |
| MM | | 0.49 | 0.26 | 0.96 | 0.51 | 0.40 | 0.38 | 0.38 |
| Mm | | 0.41 | 0.50 | 0.04 | 0.40 | 0.46 | 0.47 | 0.47 |
| mm | | 0.10 | 0.24 | 0.00 | 0.09 | 0.14 | 0.15 | 0.15 |
| M allele | | 0.69 | 0.51 | 0.98 | 0.71 | 0.63 | 0.61 | 0.61 |
| m allele | | 0.31 | 0.49 | 0.02 | 0.29 | 0.37 | 0.39 | 0.39 |
| *p*_HWE_-value | | 0.2328 | 0.9095 | 0.3748 | 0.3888 | 0.8378 | 0.9823 | 0.8305 |

Table S2 Differences on the adiponectin level in KCP-II based on the genotype combination between rs3865188- rs2241767, rs3865188- rs382179 and rs3865188- rs6773957.

| *CDH13* | *APN* | Adiponectin levels | | | |  |
| --- | --- | --- | --- | --- | --- | --- |
|  |  | MM/MM | | mm/mm | |  |
|  |  | mean±std err | median | mean±std err | median | *p*-value |
| *rs3865188* | *rs2241767* | 9.633±0.447 | 8.100 | 6.1890±1.067 | 5.220 | 0.0219 |
|  | *rs3821799* | 9.677±0.917 | 7.415 | 6.1658±1.125 | 4.800 | 0.0029 |
|  | *rs6773957* | 9.5015±0.430 | 8.000 | 6.1051±0.510 | 5.030 | 0.0022 |
